# Heart rate recovery after orthostatic challenge and cardiopulmonary exercise testing in older individuals: prospective multicentre observational cohort study

**DOI:** 10.1016/j.bjao.2023.100238

**Published:** 2023-11-03

**Authors:** Aaron James, David Bruce, Nicholas Tetlow, Amour B.U. Patel, Ethel Black, Nicole Whitehead, Anna Ratcliff, Alice Jamie Humphreys, Neil MacDonald, Gayle McDonnell, Ravishankar Raobaikady, Jeeveththaa Thirugnanasambanthar, Jeuela I. Ravindran, Nicole Whitehead, Gary Minto, Tom E.F. Abbott, Shaman Jhanji, Don Milliken, Gareth L. Ackland

**Affiliations:** 1 Department of Anaesthesia, University Hospitals Plymouth NHS Trust, Plymouth, UK; 2 Department of Anaesthesia, Perioperative Medicine and Critical Care, Royal Marsden Hospital, London, UK; 3 Translational Medicine and Therapeutics, William Harvey Research Institute, Queen Mary, University of London, UK; 4 Department of Anaesthesia and Perioperative Medicine, Royal London Hospital, London, UK

**Keywords:** exercise, heart rate recovery, orthostatic, prognostic

## Abstract

**Background:**

Impaired vagal function in older individuals, quantified by the ‘gold standard’ delayed heart rate recovery after maximal exercise (HRR^exercise^), is an independent predictor of cardiorespiratory capacity and mortality (particularly when HRR ≤12 beats min^−1^). Heart rate also often declines after orthostatic challenge (HRR^orthostatic^), but the mechanism remains unclear. We tested whether HRR^orthostatic^ reflects similar vagal autonomic characteristics as HRR^exercise^.

**Methods:**

Prospective multicentre cohort study of subjects scheduled for cardiopulmonary exercise testing (CPET) as part of routine care. Before undergoing CPET, heart rate was measured with participants seated for 3 min, before standing for 3 min (HRR^orthostatic^). HRR^exercise^ 1 min after the end of CPET was recorded. The primary outcome was the correlation between mean heart rate change every 10 s for 1 min after peak heart rate was attained on standing and after exercise for each participant. Secondary outcomes were HRR^orthostatic^ and peak VO_2_ compared between individuals with HRR^exercise^ <12 beats min^−1^.

**Results:**

A total of 87 participants (mean age: 64 yr [95%CI: 61–66]; 48 (55%) females) completed both tests. Mean heart rate change every 10 s for 1 min after peak heart rate after standing and exercise was significantly correlated (*R*^2^=0.81; *P*<0.0001). HRR^orthostatic^ was unchanged in individuals with HRR^exercise^ ≤12 beats min^−1^ (*n*=27), but was lower when HRR^exercise^ >12 beats min^−1^ (*n*=60; mean difference: 3 beats min^−1^ [95% confidence interval 1–5 beats min^−1^]; *P*<0.0001). Slower HRR^orthostatic^ was associated with lower peak VO_2_ (mean difference: 3.7 ml kg^-1^ min^−1^ [95% confidence interval 0.7–6.8 ml kg^-1^ min^−1^]; *P*=0.039).

**Conclusion:**

Prognostically significant heart rate recovery after exhaustive exercise is characterised by quantitative differences in heart rate recovery after orthostatic challenge. These data suggest that orthostatic challenge is a valid, simple test indicating vagal impairment.

**Clinical trial registration:**

researchregistry6550.

Delayed heart rate recovery after exercise has repeatedly been shown to precede cardiovascular events and death, with values <12 beats min^−1^ particularly strongly associated with poorer outcomes.^1^, ^2^, ^3^, ^4^, ^5^ Heart rate recovery after exercise is the gold standard prognostic measure for assessing cardiac vagal dysregulation, as evidenced by pharmacological blockade of vagal reactivation with atropine abolishing heart rate recovery after exercise.^6^ In individuals with heart failure, the heart rate recovery after exercise is blunted, in contrast to the rapid decline observed in well-trained athletes.^6^ Preoperative impairment of cardiac vagal activity, as quantified by cardiopulmonary exercise testing (CPET), is also an independent predictor of perioperative myocardial injury.^6^

The autonomic nervous system also coordinates a rapid physiological response to orthostasis, by integrating afferent signals from the skeletal muscle pump, and the arterial and cardiopulmonary baroreflexes.^7^ The heart rate typically increases 10 s after standing to counteract the gravitational forces acting on blood pressure^8^ through cardiac vagal withdrawal.^7^ Thereafter, heart rate declines as arterial blood pressure rebounds, which is often steepest 10–20 s after peak heart rate is attained.^9^ An attenuated heart rate recovery response after an orthostatic challenge may reflect dysregulation of the parasympathetic (vagal) limb of the autonomic nervous system, particularly with frailty and cardiometabolic comorbidity.^10^ However, this hypothesis has not been formally tested.

Establishing a direct link between heart rate recovery after orthostatic and exercise tests would add further value in understanding the mechanisms of disease involving autonomic dysfunction at scale in older populations. Moreover, advances in molecular neuroscience,^11^ physiological modelling of the human exercise response,^12^ and genome-wide association studies^13^^,^^14^ suggest that the strength of cardiac vagal activity causally determines exercise capacity. Thus, using a simpler standardised method to quantify vagal function may help identify individuals who have impaired cardiorespiratory exercise capacity.^15^ Here, we hypothesised that individuals with delayed heart rate recovery after exercise also exhibit slower heart rate recovery after standing (Fig 1). In particular, we aimed to establish whether the prognostically significant value of delayed heart rate recovery after exercise ≤12 beats min^−1^ was associated with different orthostatic recovery profiles.

**Fig 1:**
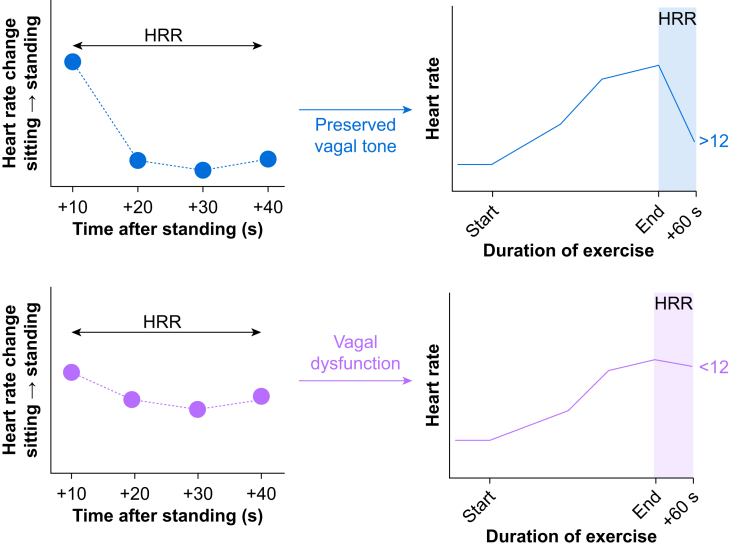
Study hypothesis, designed to establish whether there is equivalence between orthostatic and exercise heart rate recovery profiles in indicating cardiac vagal impairment. Illustration highlights identifying individuals from orthostatic testing who are likely to have heart rate recovery (HRR) after exercise ≤12 beats min^−1^, a strongly prognostic threshold.

## Methods

### Study design

This multicentre, prospective, observational cohort study was approved by the HRA and Health and Care Research Wales Research Ethics Committee (MREC: 19/SC/0656) on 12 February 2020 and conducted in accordance with the principles of the Declaration of Helsinki, the Research Governance Framework and The Strengthening the Reporting of Observational Studies in Epidemiology (STROBE) guideline (Supplementary material). The study was prospectively registered (researchregistry6550) on 8 February 2021. Written informed consent was obtained from all patients before CPET.

### Study setting

This study was undertaken at preoperative exercise testing facilities at The Royal London Hospital (Barts Health NHS Trust), The Royal Marsden Hospital, and University Hospitals Plymouth NHS Trust. Recruitment and data collection took place from 26 October 2021 to 24 February 2022.

#### Study participants

Participants aged ≥50 yr referred for CPET for medical or preoperative assessment were eligible. Exclusion criteria were refusal or incapacity to provide written informed consent, inability to stand, or the presence of absolute contraindications to undertaking CPET.

### Heart rate measurement

The heart rate was measured using the same continuous 12-lead ECG monitoring for both orthostatic and CPET protocols and averaged over each 3-s period.

### Orthostatic challenge

Participants first rested comfortably in a chair for 3 min before proceeding to stand for 3 min, in a quiet room at controlled ambient temperature. They were instructed to stand up as quickly as possible, typically in <5 s. In this population no assistance was required. The zero time point for each individual was set by research personnel at the point where the participant began to rise from the chair. The heart rate recovery after orthostatic challenge (HRR^ortheostatic^) was defined as the change in heart rate from the peak heart rate to the heart rate 60 s after peak heart rate was attained.

### Cardiopulmonary exercise testing

Once the standing period was completed, participants proceeded to symptom-limited CPET using electromagnetically braked cycle ergometers.^16^ A standardised incremental ramp protocol was used in each centre, as published previously.^16^ The heart rate recovery after exercise (HRR^exercise^) during the first minute of the recovery period was calculated as the difference between heart rate at the end of the incremental exercise and heart rate after 1 min of the recovery period. Clinicians and participants were masked to the results of orthostatic heart rate recovery. Independent analysers compiled the orthostatic challenge data masked to exercise heart rate recovery data. HRR^exercise^ defined two heart rate recovery endotypes: >12 and ≤12 beats min^−1^. Heart rate recovery ≤12 beats min^−1^ after exercise has repeatedly been shown to precede cardiovascular events and death.^1^, ^2^, ^3^, ^4^, ^5^

### Primary outcome

The primary outcome was the correlation between mean heart rate change every 10 s for 1 min after peak heart rate was attained on standing and after exercise for each participant. The slopes of heart rate decline from peak heart rate to 60 s were also compared between HRR^orthostatic^ and HRR^exercise^ by Fisher's *r*-to-*z* transformation, presented as proportions of participants with a statistically non-significant difference when comparing gradients of decline in heart rate for HRR^orthostatic^ and HRR^exercise^.

### Secondary outcomes

1. Absolute difference in HRR^orthostatic^ compared between participants with HRR^exercise^ <12 *vs* HRR^exercise^ >12 beats min^−1^.
2. Time to reach peak heart rate after standing up.
3. Peak heart rate after standing up.
4. Peak VO_2_ and resting heart rate compared between categorical orthostatic heart rate recovery, as dichotomised by HRR^exercise^ ≤12 or >12 beats min^−1^.

### Statistical analysis

We used NCSS 2021 (NCSS, Kaysville, UT, USA) to analyse the data. Heart rate recovery at 1 min after the end of incremental exercise was dichotomised according to a threshold of ≤12 beats min^−1^. Recovery values for heart rate were binned in 10 s epochs for the 60 s periods after attainment of peak heart rate after orthostatic challenge and at the end of exercise. Data are presented as median (inter-quartile range). Binary data are presented as percentages.

For the primary outcome, having confirmed monotonicity of the variables examined, Spearman's correlation coefficient was used to assess mean heart rate change every 10 s for 1 min after peak heart rate was attained on standing and after exercise for each participant (Supplementary Fig. S1). Fisher's r-to-z transformation^17^ for Spearman's correlation coefficients determined for heart rate recovery after standing and after exercise was used to examine whether there were any differences in the slope correlation coefficients between heart rate after standing and exercise^18^; *P*<0.01 was used as the threshold to define an intra-individual difference in heart rate recovery characteristics, which reduces the risk of incorrectly rejecting the null hypothesis at a *P*-value threshold of 0.05 when using Fisher's method.^19^

For secondary outcomes, repeated measures analysis of variance (anova) was used to analyse absolute changes in heart rate over 60 s binned in 10 s (time × HRR^exercise^ endotype). We also examined whether HRR^orthostatic^ threshold values identified in individuals with HRR^exercise^ ≤12 beats min^−1^ were associated with lower cardiorespiratory exercise capacity. For all comparisons, we adjusted for age as a continuous covariate (general linear measures anova). Statistical significance was set for other comparisons at P≤0.05.

### Sample size calculation

We estimated that correlation between the autonomic response to standardised orthostatic challenge (sitting to standing) against the ‘gold standard’ exercise-evoked heart rate recovery required 92 participants to detect a significant correlation between orthostatic and exercise heart rate recovery in each individual, where *r*≥0.7; α=0.01; 1–β=0.9; PASS 12, NCSS).

## Results

### Study characteristics

Three centres recruited 92 participants undergoing CPET for medical or preoperative evaluation (Table 1). A total of 87 participants (median age: 63 yr [57–72]; 48 [55%] females) completed both tests. Data were not obtained from five individuals because of poor quality heart rate recordings. Sixteen (18%) participants were taking cardiovascular medications, with 28% (*n*=24) having received a diagnosis related to cardiorespiratory disease (Table 1). The mean (standard deviation) resting heart rate before exercise was 75 beats min^−1^ (14). After standing, 15 (95% confidence interval [CI] 14–16) measures of heart rate were made every 3 s during the 60 s after peak heart rate after standing. During CPET, the mean peak heart rate was 141 (22) beats min^−1^, which declined to 126 beats min^−1^ (20) 60 s after the end of exercise. The mean peak VO_2_ was 18.5 (6.6) ml kg^–1^ min^−1^. There were no adverse events reported after either orthostatic or exercise tests.

**Table 1:** Participant characteristics. All data presented as *n* (%); mean (standard deviation). Subjects were divided into two groups on the basis of heart rate recovery (HRR) after exercise, with 27 subjects having HRR≤12 beats min^−1^ after exercise, compared with 60 subjects having HRR>12 beats min^−1^. Age was distributed normally (Shapiro–Wilk normality test value: 0.99; *P*-value: 0.65). COPD, chronic obstructive pulmonary disease. ∗HRR data were not obtained from five individuals because of poor quality heart rate recordings.

	All subjects (*n*=92)	HRR ≤12 (*n*=27)∗	HRR ≥12 (*n*=60)∗
Female gender	43 (46)	12 (44)	28 (47)
Age (yr)	64 (10)	68 (11)	61 (9)
American Society of Anesthesiology physical status ≥2	52 (56)	19 (70)	29 (48)
Chronic comorbid disease			
Hypertension	17 (18)	9 (33)	6 (10)
Ischaemic heart disease	4 (4)	1 (4)	3 (5)
Cardiac failure	2 (2)	0 (0)	2 (3)
Diabetes mellitus	6 (7)	2 (7)	4 (7)
COPD/pulmonary disease	8 (9)	5 (19)	2 (3)
Preoperative medication			
Beta-blocker	6 (7)	2 (7)	4 (7)
Calcium channel antagonist (dihydropyridines)	6 (7)	4 (15)	1 (2)
ACE inhibitor/angiotensin II receptor blocker	10 (11)	5 (19)	3 (5)
Other antihypertensive	6 (7)	1 (4)	4 (7)

### Primary outcome: correlation between decline from peak heart rate after standing and exercise

**Fig 2:**
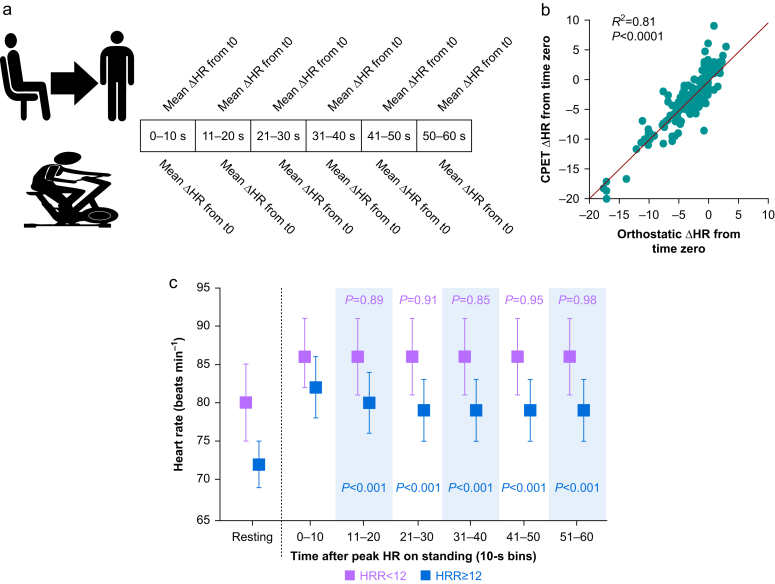
Primary and secondary outcomes for heart rate recovery. (a) Method of analysis: the decline in mean heart rate (HR) was calculated for each 10-s bin for 60 s after peak heart rate was attained on standing and CPET. Mean HR was then correlated for each bin for every participant between standing and CPET. (b) Correlation between mean HR decline in each 10-s bin from time zero, defined as the time at which peak heart rate occurred on standing and during CPET. (c) Orthostatic challenge mean (95% confidence interval [CI]) heart rates according to HRR^exercise^ response > or <12 beats min^−1^. *P*-values refer to within group comparisons (Tukey–Kramer *post hoc* test), comparing mean heart rate over 60 s in 10-s bin *vs* peak heart rate. Blue *P*-values refer to within HRR^exercise^ >12; purple *P*-values refer to within HRR^exercise^ <12. The mean difference in HR^ortho^ between HRR^exercise^ groups was 3 beats min^−1^ (95% CI 1–5); F_(5,401)_=6.68; *P*<0.001). CPET, cardiopulmonary exercise testing; HRR, heart rate recovery.

Mean heart rate decline compared every 10 s for 1 min after orthostatic challenge and peak exercise (Fig 2a) was significantly correlated (*R*^2^=0.81; *P*<0.0001; Fig 2b). Summary data for *r* values for the slopes of heart rate change after HRR^orthostatic^ and HRR^exercise^ are provided in Table 2. Some 43/87 (49%) of participants did not have statistically significantly different HRR slopes for HRR^orthostatic^ and HRR^exercise^.

**Table 2:** Primary outcome: *R* values for both HRR^orthostatic^ and HRR^exercise^. HRR, heart rate recovery; sd, standard deviation.

	Correlation coefficients different by z-test					Correlation coefficient not different by z-test				
	Mean	SD	Minimum	Maximum	Range	Mean	sd	Minimum	Maximum	Range
Orthostatic HRR	−0.83	0.34	−0.99	0.75	1.75	−0.80	0.25	−1.00	0.09	1.08
Exercise HRR	−0.14	0.67	−0.96	0.98	1.93	−0.73	0.35	−0.98	0.92	1.90

### Secondary outcomes

Absolute difference in HRR^orthostatic^ compared between participants with HRR^exercise^ <12 vs HRR^exercise^ >12 beats min^−1^

**Fig 3:**
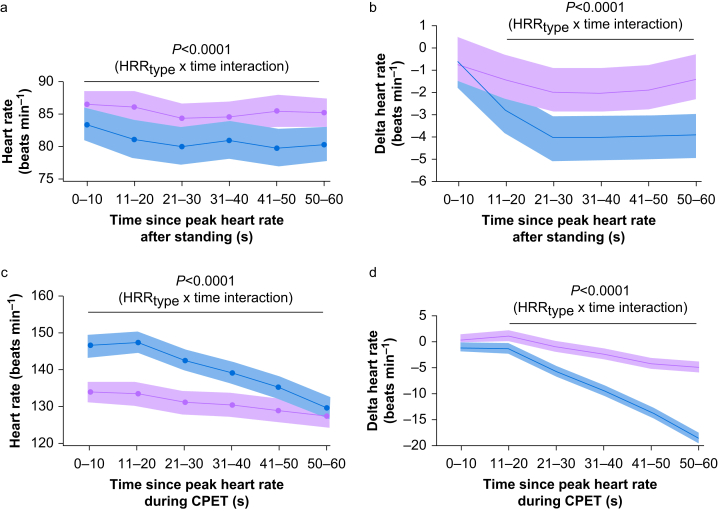
Sequential changes in absolute and delta heart rate from peak values attained in CPET and orthostatic challenge. (a) Absolute heart rate values after attaining peak heart rate after standing. Blue and purple lines/shading shown according to HRR^exercise^ response >12 beats min^−1^ (blue lines/shading) or ≤12 beats min^−1^ (purple lines shading). *P*-value refers to between group comparisons (Tukey–Kramer *post hoc* test), comparing mean heart rate over 60 s in each 10 s bin between individuals with HRR^exercise^ >12 *vs* those with HRR^exercise^ ≤12. (b) Individualised mean differences (95% confidence interval [CI]) in heart rate (delta heart rate) from peak heart rate after standing, shown over sequential 10 s bins for 60 s after attaining peak heart rate. Data are shown according to HRR^exercise^ response >12 beats min^−1^ (blue lines/shading) or ≤12 beats min^−1^ (purple lines shading). The mean absolute delta change in HR^ortho^ was 3 beats min^−1^ (95% CI 2–4) *P*<0.0001) for individuals with HRR^exercise^ response >12 beats min^−1^. For individuals with HRR^exercise^ response ≤12 beats min^−1^, the mean absolute delta change in HR^ortho^ was 1 beat min^−1^ (95% CI −1 to 2); *P*=0.67). *P*-values refer to between group comparisons (Tukey–Kramer *post hoc* test), comparing mean heart rate over 60 s in each 10-s bin between individuals with HRR^exercise^ >12 *vs* those with HRR^exercise^ ≤12. (c) Absolute heart rate values after attaining peak heart rate in CPET. Blue and red lines/shading shown according to HRR^exercise^ response >12 beats min^−1^ (blue lines/shading) or ≤12 beats min^−1^ (purple lines shading). The mean difference in HR between HRR^exercise^ groups was 9 beats min^−1^ (95% CI 1–18); F_(5,409)_=7.99; *P*<0.0001). Absolute heart rate was higher at all time points during the 60 s recovery after peak exercise for individuals who subsequently had HRR^exercise^ >12 beats min^−1^ after recovery from peak heart rate in CPET (*P*<0.0001; time × HRR^exercise^ interaction). (d) Individualised mean differences (95% CI) in heart rate (delta heart rate) for 60 s after attaining CPET peak heart rate in sequential 10 s bins. Data are shown according to HRR^exercise^ response >12 beats min^−1^ (blue lines/shading) or ≤12 beats min^−1^ (purple lines shading). The mean difference in delta HR between HRR^exercise^ groups was 6 beats min^−1^ (95% CI 4–8); F_(5,409)_=7.70; *P*<0.0001). *P*-values refer to between group comparisons (Tukey–Kramer *post hoc* test), comparing mean heart rate over 60 s in each 10-s bin between individuals with HRR^exercise^ >12 *vs* those with HRR^exercise^ ≤12. CPET, cardiopulmonary exercise testing; HRR, heart rate recovery.

The mean absolute change in heart rate from peak heart rate 60 s after standing was 3 beats min^−1^ (95% CI 2–4) *P*<0.0001) for individuals with HRR^exercise^ response >12 beats min^−1^ (Fig 2c). For individuals with HRR^exercise^ response ≤12 beats min^−1^, the mean absolute change in in heart rate 60 s after standing was 1 beat min^−1^ [95% CI −1 to 2]; *P*=0.67). Sequential changes in absolute and heart rate decline from peak values attained in CPET and orthostatic challenge are shown in Fig 3.

### Time to reach peak heart rate after standing up

The time to reach peak heart rate after standing up was not different between the two HRR^exercise^ groups (mean difference between HRR^exercise^ >12 *vs* ≤12: 2 s (95% CI −9 to 11); *P*=0.748).

### Peak heart rate after standing up

The peak heart rate also did not differ between participants with, or without, HRR^exercise^ >12 (mean difference: 5 beats.min^−1^ (95% CI −1 to 11); *P*=0.097).

Peak VO_2_ and resting heart rate compared between categorical orthostatic heart rate recovery, as dichotomised by HRR^exercise^ ≤12 or >12 beats min^−1^

**Fig 4:**
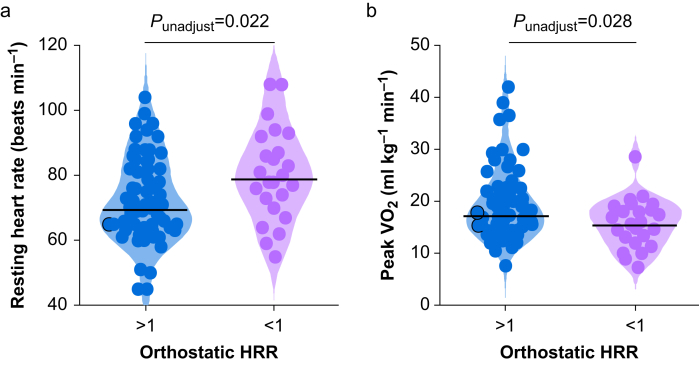
Cardiopulmonary exercise test measures, stratified by orthostatic heart rate decline. Violin plots demonstrating that absent HRR^orthostatic^ (recovery over 60 s after peak heart rate on standing) was associated with higher resting heart rate (mean difference: 8 beats min^−1^ (95% confidence interval [CI] 2–14); *P*=0.019) and lower peak VO_2_ (mean difference: 3.7 ml kg^–1^ min^−1^ (95% CI 0.7–6.8); *P*=0.039), controlling for age. Unadjusted *P*-values are shown in each panel; black lines represent median values. HRR, heart rate recovery.

An HRR^orthostatic^ ≥1 beats min^−1^ was associated with higher resting heart rate (mean difference: 8 beats min^−1^ (95% CI 2–14); *P*=0.019; Fig 4a) and lower peak VO_2_ (mean difference: 3.7 ml kg^–1^ min^−1^ (95% CI 0.7–6.8); *P*=0.039; Fig 4b), controlling for age.

## Discussion

Our study identifies that alterations in cardiac vagal autonomic balance are common to both orthostatic challenge and exercise, both of which have been useful predictors of mortality at the population level.^20^^,^^21^ The lack of quantitative change in orthostatic heart rate recovery in individuals with impaired heart rate recovery after exercise suggests that this orthostatic challenge may serve as a useful screening tool to identify individuals with the most profound, prognostically relevant vagal dysautonomia. Furthermore, given that standing from sitting in a chair can usually be readily performed by functionally mobile individuals, these data suggest that such an orthostatic challenge is a useful surrogate for uncovering vagal dysfunction which otherwise would require more involved autonomic stressors such as formal CPET. Our highly scalable test is cost-effective (minimal equipment is required), rapidly reproducible (it can be conducted almost anywhere), noninvasive, and simple to set up and conduct for both the patient and clinician.

The Irish Longitudinal Study on Ageing (TILDA) has demonstrated that simple measures of heart rate in response to standing has prognostic values for a range of outcomes in the older adult population.^9^^,^^22^^,^^23^ The speed of heart rate recovery after an orthostatic challenge predicts mortality and distinguishes that risk between different age groups and individuals with existing cardiovascular disease.^21^ An attenuated heart rate recovery to active standing has long been postulated to reflect dysregulation of the parasympathetic nervous system, as parasympathetic inhibition appears to account for the small increase in heart rate after standing. Heart rate recovery after standing has been attributed to parasympathetic reactivation, but direct comparisons between the heart rate recovery response to orthostatic challenge and exercise have not been systematically examined. Developing a simpler, standardised orthostatic test that is readily performed and robustly maps to exercise-evoked heart rate recovery may have considerable impact on assessing autonomic function in large populations.

In parallel with heart rate recovery after cessation of exercise, a slower speed of heart rate recovery 10–20 s after standing (at which point peak heart rate is normally achieved) is associated with a higher risk of long-term mortality. In the general population of individuals aged >50 yr, slow heart rate recovery after orthostatic challenge is associated with shortened life expectancy.^24^ These data suggest that preoperative cardiac vagal dysfunction identified from orthostatic manoeuvres may increase the risk of postoperative morbidity and mortality after noncardiac surgery. Some studies suggest that serial and dynamic measures showing loss of vagal activity (obtained from heart rate recovery and time- and frequency-domain measures of heart rate variability after supine to head-up orthostatic challenge pre- and post-noncardiac surgery) is associated with perioperative myocardial injury and potential noncardiac morbidity.^24^^,^^25^ The orthostatic challenge seems to bear a close relationship with other autonomic measures. However, overall, there is a paucity of data as to whether orthostatic manoeuvres can elicit cardiac vagal dysfunction relevant to the perioperative period and its associated risks. Further studies are required to determine the autonomic changes from orthostatic testing that characterise a small but significant population of higher-risk surgical patients, and the relationship of these changes to morbidity outcomes. Given the short duration of the test, the active stand is only useful for the detection of immediate and classical orthostatic heart rate responses, with its diagnostic value yet to be proved. Nonetheless, the applications of orthostatic testing augment CPET data, bringing monitoring of real-time autonomic physiological changes to the bedside. Disruption of autonomic control is robustly and independently associated with the perioperative period, and orthostatic testing can elicit these changes, which are otherwise often symptomatically and clinically undetectable.

A strength of this prospective multicentre study was the intra-individual comparison made between the two autonomic challenges conducted consecutively. Masking of the heart rate recovery data was rigorous, with clinicians and participants masked to the results of orthostatic heart rate recovery and independent analysers compiling the orthostatic challenge data masked to exercise heart rate recovery data. Mapping the orthostatic challenge thresholds to measures of cardiorespiratory capacity adds further insight. The findings from each centre were concordant. A limitation is that these comparisons were made in individuals referred for CPET for medical reasons, reducing the generalisability of the findings.

In summary, prognostically significant heart rate recovery after exhaustive exercise is characterised by quantitative differences in heart rate recovery after orthostatic challenge. These data suggest that orthostatic challenge is a valid, simple test indicative of vagal impairment. Simplifying preoperative assessment through implementation of orthostatic testing and rationing of CPET resources may benefit perioperative medicine. Additional work is required to establish whether orthostatic heart rate recovery provides similar predictive data on perioperative morbidity compared with heart rate recovery after exercise.^16^^,^^26^^,^^27^ To that end, our ongoing randomised controlled trial examining the impact of targeted heart rate control on perioperative myocardial injury (ISRCTN12903789) is incorporating orthostatic heart rate recovery to phenotype the autonomic characteristics of patients before surgery.

## Authors’ contributions

Designed the study protocol, statistical design: ABUP, GLA

Recruitment and intervention: ABUP, GLA, AJ, DB, NT, NM, EB, NW, AR, AJH, GM, RR, NW, GM, SJ, DM

Data extraction: AJ, DB, NT, NM

Masked orthostatic challenge/CPET data analysis: JT, JIR

Statistical analysis: GLA, TEFA

Contributed to the first manuscript draft and subsequent revisions: all authors

## Declarations of interest

GLA: editor and editorial board member of the British Journal of Anaesthesia and BJA Open; consultancy work for GlaxoSmithKline, unrelated to this work.

## Funding

National Institute for Health and Care Research (NIHR) Advanced Fellowship (NIHR300097 to GLA); British Heart Foundation programme grant (RG/19/5/34463). UK NIHR CLRN Portfolio research support.
