# Supplementary material for: Heart rate recovery after orthostatic challenge and cardiopulmonary exercise testing in older individuals: prospective multicentre observational cohort study
Source: BJA Open. 2023 Nov 3;8:100238. doi: 10.1016/j.bjao.2023.100238 (PMC10654531; doi:10.1016/j.bjao.2023.100238)

**Supplementary information**

**Heart rate recovery after orthostatic challenge and cardiopulmonary exercise testing in older individuals: prospective multicentre observational cohort study.**

Aaron James,^1*^ David Bruce,^2*^ Nicholas Tetlow,^2*^ Amour B. U. Patel,^3*^ Ethel Black,^2^ Nicole Whitehead,^2^ Anna Ratcliff,^1^ Alice Jamie Humphreys,^1^ Neil MacDonald,^4^ Gayle McDonnell,^4^ Ravishankar Raobaikady,^3^ Jeeveththaa Thirugnanasambanthar,^3^ Jeuela I. Ravindran,^3^ Nicole Whitehead,^2^ Gary Minto,^1^ Shaman Jhanji,^2^ Don Milliken,^2^ Gareth L. Ackland ^3,4^

Contents

[Supplementary Figure 1. Standardised comparisons made to construct correlation in heart rate recovery between orthostatic challenge and exercise testing. 2](#_Toc129004082)

# Supplementary Figure 1. Standardised comparisons made to construct correlation in heart rate recovery between orthostatic challenge and exercise testing.

Stylised example for one individual.


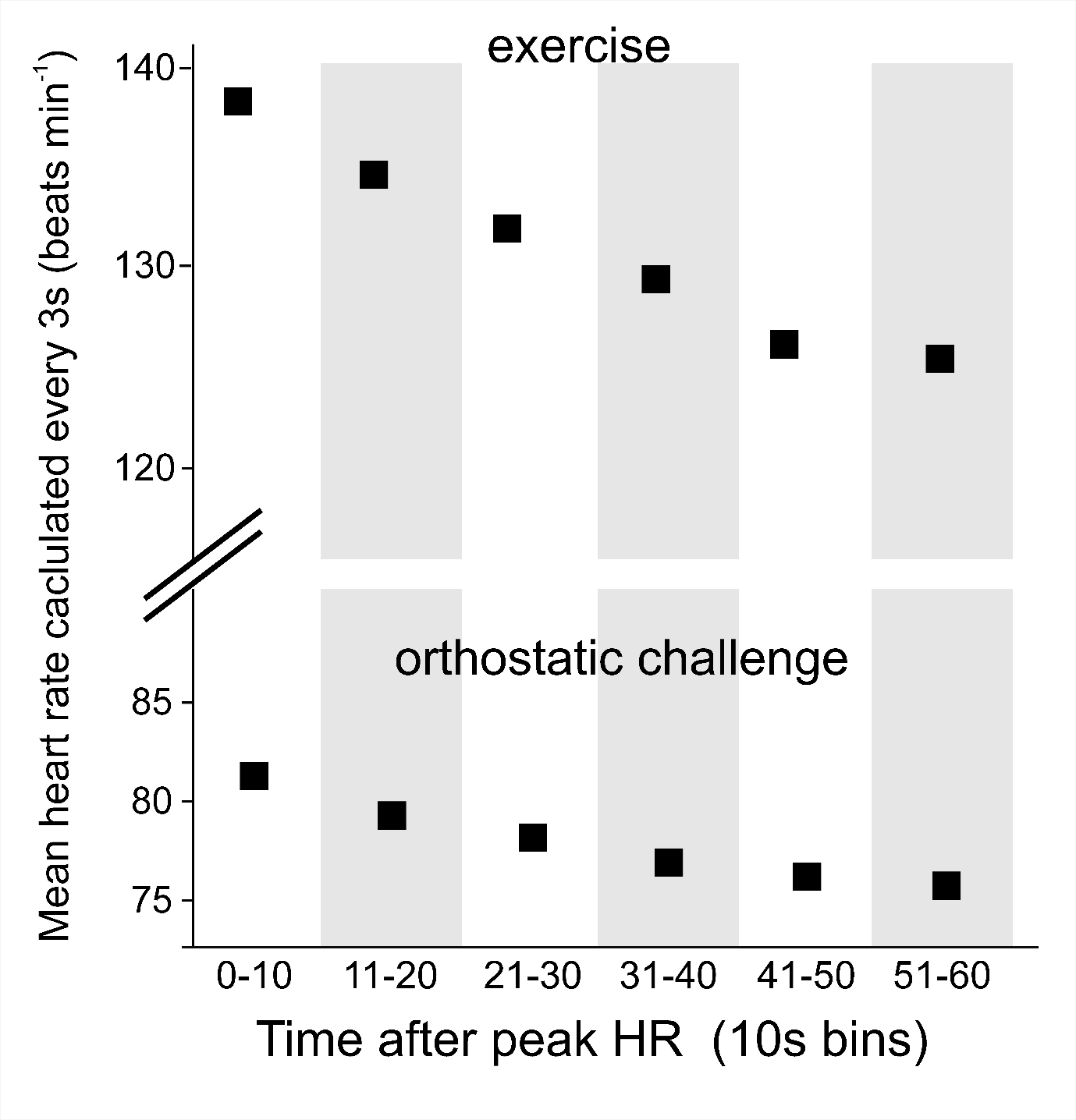

Supplement: Multimedia component 1 [file mmc1.docx]
